# Mass spectrometry based identification of AMP‐O‐Tris generated by *Thermococcus onnurineus* Cas10

**DOI:** 10.1002/2211-5463.70235

**Published:** 2026-03-23

**Authors:** Su‐Jin Lee, Ga Seul Lee, JinHan Kim, Kwang‐Hyun Park, Seong‐Ryung Go, Jeong Hee Moon, Eui‐Jeon Woo

**Affiliations:** ^1^ Bio‐Design & Editing Research Center Korea Research Institute of Bioscience & Biotechnology (KRIBB) Daejeon Republic of Korea; ^2^ Core Research Facility & Analysis Center Korea Research Institute of Bioscience & Biotechnology (KRIBB) Daejeon Republic of Korea; ^3^ College of Pharmacy Chungbuk National University Cheongju Republic of Korea; ^4^ Department of Proteome Structural Biology, KRIBB School of Bioscience University of Science and Technology (UST) Daejeon Republic of Korea; ^5^ Critical Diseases Diagnostics Convergence Research Center Korea Research Institute of Bioscience and Biotechnology (KRIBB) Daejeon Republic of Korea

**Keywords:** adenylylation, AMP‐O‐Tris, Cas10, cyclic oligoadenylate, mass spectrometry, noncanonical enzymatic activity, Type III CRISPR–Csm

## Abstract

Cas10, the catalytic core of type III CRISPR–Csm systems, synthesizes cyclic oligoadenylate (cOA) second messengers to activate downstream immune responses. Although Cas10 activity is regulated by complex assembly, the nucleophile selectivity and off‐pathway reactivity of isolated Cas10 remain poorly understood. Here, using HPLC separation and subsequent tandem mass spectrometry (MS/MS) analysis, we identify and structurally characterize AMP‐O‐Tris as a noncanonical adenylylated product generated by isolated *Thermococcus onnurineus* Cas10. Our results reveal that purified Cas10 exhibits relaxed nucleophile selectivity, diverting ATP turnover into nonproductive adenylylation involving buffer‐derived nucleophiles. This suggests that effector complex assembly constrains Cas10 reactivity to promote efficient cOA synthesis and suppress off‐pathway chemistry. Furthermore, interception of reactive intermediates by buffer‐derived nucleophiles may represent a potential chemical fail‐safe that limits unintended signaling when Cas10 is uncoupled from the complex. Together, our study provides mechanistic insight into Cas10 regulation and informs the development of robust type III‐based diagnostic platforms.

**Impact statement**

Our study reveals that Cas10 exhibits latent catalytic flexibility when isolated, identifying a noncanonical adenylation reaction. These findings demonstrate how complex assembly constrains enzymatic specificity to prevent aberrant signaling. This mechanistic insight is crucial for improving the fidelity and design of next‐generation CRISPR‐based diagnostic platforms.

## Graphical abstract

Isolated *Thermococcus onnurineus* Cas10 generates the noncanonical ATP‐derived product AMP‐O‐Tris while in Tris‐containing buffer as identified via mass spectrometry, revealing relaxed nucleophile selectivity under isolated conditions. These findings suggest that multiprotein Csm complex assembly restricts Cas10 reactivity toward canonical cyclic oligoadenylate (cOA) synthesis while limiting off‐pathway adenylylation, thereby contributing to catalytic fidelity in type III CRISPR systems.

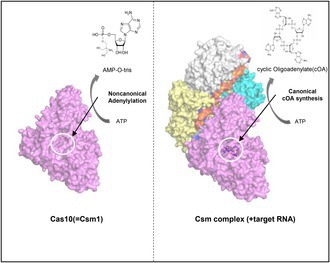

**Abbreviations**
CID: collision‐induced dissociation
cOA: cyclic oligoadenylate
HPLC: high‐performance liquid chromatography
MS: mass spectrometry
MS/MS: tandem mass spectrometry

Type III CRISPR–Csm systems provide adaptive immunity in bacteria and archaea through RNA‐guided interference mechanisms coupled to nucleotide‐based signaling pathways [1, 2]. These systems assemble multisubunit effector complexes that recognize target RNA and trigger coordinated RNA cleavage, DNA targeting, and second‐messenger signaling [3, 4, 5]. Genetic and biochemical studies have shown that dysregulation of these activities can lead to autoimmunity, underscoring the need for tight regulatory control of effector functions [3, 4, 6].

At the core of these complexes is Cas10 (also referred to as Csm1 in type III‐A systems), a multidomain enzyme that catalyzes ATP‐dependent adenylylation reactions to generate cyclic oligoadenylate (cOA) molecules [7, 8, 9]. These nucleotide‐derived second messengers activate downstream effector proteins, such as CARF‐domain ribonucleases, including Csm6, thereby amplifying antiviral responses [7, 8, 9]. The role of Cas10 as a central hub linking RNA sensing to nucleotide‐based signaling is now well established across diverse type III CRISPR systems [7, 8, 9, 10].

Biochemical and structural studies have demonstrated that binding of target RNA to the Csm effector complex activates the Palm polymerase domains of Cas10, leading to cOA synthesis [7, 8, 11]. Notably, structural and biochemical analyses of the Thermococcus onnurineus Csm complex demonstrated that the assembled effector predominantly produces cOA4 together with detectable levels of cOA3, providing a defined baseline for canonical Cas10‐dependent signaling in this organism [12]. The resulting cOA molecules allosterically activate ancillary nucleases, including Csm6, which mediate collateral RNA degradation as part of the immune response [7, 8, 9]. To prevent excessive or prolonged activity, cOA signaling is tightly regulated by dedicated ring nucleases that degrade cyclic oligoadenylates and terminate signaling [13, 14]. Together, these findings highlight the importance of precise control over Cas10‐derived nucleotide chemistry within assembled CRISPR effector complexes [9, 10, 13, 14].

Cas10 activity is consequently widely recognized as being strictly governed by its integration into multiprotein Csm assemblies, which ensure appropriate substrate recognition and enforce canonical reaction pathways [9, 10]. Structural analysis of the Csm1 subunit from *Thermococcus onnurineus* has revealed a multidomain architecture, including an N‐terminal HD domain with single‐stranded DNA‐specific nuclease activity, underscoring the catalytic versatility of Cas10 family proteins while emphasizing the role of complex assembly in shaping their functional output [15]. Subsequent cryo‐EM and crystallographic studies have further shown that RNA‐mediated activation induces coordinated conformational rearrangements across the Csm complex, directly coupling RNA binding to Cas10 activation [16, 17].

Despite these advances, the intrinsic catalytic behavior of Cas10 in isolation, particularly with respect to nucleophile selection and adenylylation specificity, remains to be fully elucidated. Recent systematic and biochemical analyses have demonstrated that isolated Cas10 proteins exhibit little or no detectable cyclic oligoadenylate synthesis in the absence of the full surveillance complex, underscoring the importance of multiprotein assembly for functional activation [18]. This is of particular importance as type III CRISPR–Cas systems have recently emerged as a powerful platform for next‐generation RNA diagnostics, where Cas10‐mediated signal amplification must be strictly linked to target recognition [19]. In this study, we examined the ATP‐dependent reaction products generated by isolated Cas10 from *T. onnurineus* under *in vitro* conditions. Using mass spectrometry‐based analyses, we identified AMP‐O‐Tris, a noncanonical adenylylated product formed through covalent linkage of AMP to the buffer component Tris, a nucleophile previously observed in nonenzymatic ATP chemistry [20]. Our results reveal latent catalytic flexibility in Cas10 when removed from its native multiprotein assembly and suggest that complex formation plays a critical role in constraining substrate selection and suppressing nonproductive side reactions in CRISPR‐associated enzymatic activity. The overall functions of the type III CRISPR–Csm complex and the position of Cas10 addressed in this study are schematically summarized in Fig. 1. These findings provide a mechanistic foundation for improving the fidelity and sensitivity of CRISPR‐based diagnostic tools.

**Fig. 1:**
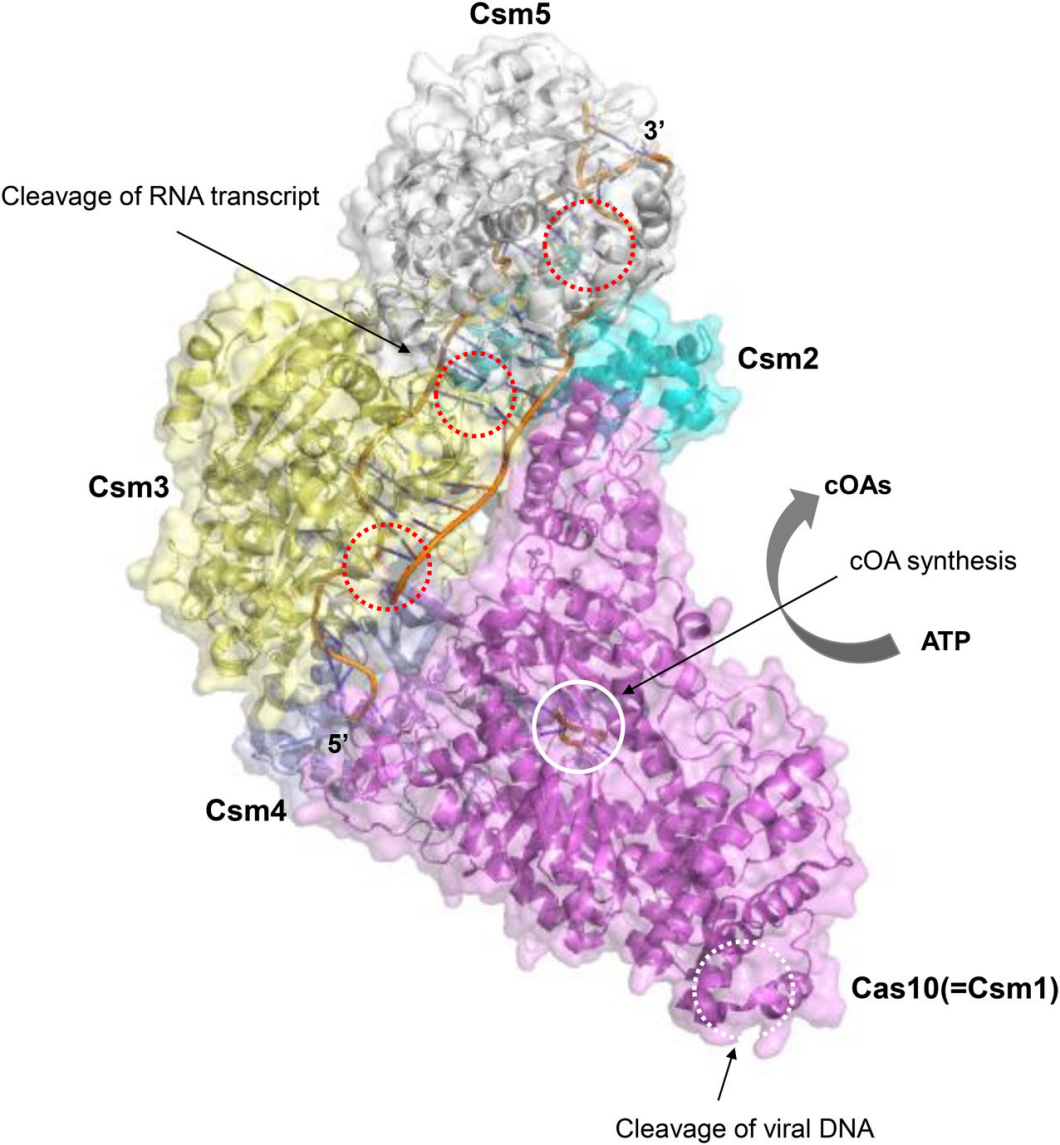
Overview of Cas10 functions within the type III CRISPR–Csm complex. Schematic representation of the type III CRISPR–Csm effector complex, highlighting RNA‐guided target recognition coupled to Cas10‐mediated ssDNA cleavage and cyclic oligoadenylate (cOA) synthesis. Structural visualization was performed using pymol based on the structure available in the RCSB Protein Data Bank.

## Materials and methods

### Protein expression and purification

Recombinant Cas10 from *T. onnurineus* was expressed in *Escherichia coli* BL21 (DE3) essentially as described previously [11]. The gene encoding Cas10 was cloned into an expression vector containing an N‐terminal His₆ tag. Protein expression was induced at mid‐log phase by addition of 0.5 mM isopropyl β‐*d*‐1‐thiogalactopyranoside, followed by incubation at 18 °C for approximately 18 h. Cells were harvested by centrifugation and resuspended in lysis buffer consisting of 30 mM Tris–HCl (pH 8.0), 500 mM NaCl, 5 mM β‐mercaptoethanol, 5% glycerol, and 1 mM phenylmethylsulfonyl fluoride (PMSF).

Cells were lysed by sonication, and insoluble material was removed by centrifugation. The clarified lysate was applied to Ni–NTA affinity chromatography resin, washed with the same lysis buffer, and Cas10 was eluted using elution buffer containing 250 mM imidazole. Eluted protein fractions were dialyzed against buffer containing 50 mM Tris/HCl (pH 8.0), 300 mM NaCl, and 5 mM β‐mercaptoethanol, followed by further purification by Superdex 200 Increase 10/300 GL. Protein purity was assessed by SDS/PAGE.

Catalytically inactive Cas10 variants carrying mutations in the conserved GGDD motif were generated by site‐directed mutagenesis and purified using the same procedure as wild‐type Cas10. The Cas10–Csm4 subcomplex was prepared by co‐expression of Cas10 and Csm4 in *E. coli* using a pRSF‐Duet expression vector, following a strategy described previously [11]. The subcomplex was purified using the same affinity chromatography and GPC procedures as described above.

### *In vitro* Cas10 reaction assays

ATP‐dependent reactions were performed by incubating purified Cas10 in Tris‐based reaction buffer, following established Cas10 nucleotide reaction assays [7, 8]. Unless otherwise indicated, reactions were assembled in a total volume of 20 μL containing 50 mM Tris/HCl (pH 8.0), 500 μM ATP, 1 μM purified Cas10, and divalent metal ions (NiCl_2_ or ZnCl_2_ at millimolar concentrations). Reactions were incubated at 55 °C for up to 24 h. Control reactions were performed either in the absence of enzyme or using catalytically inactive Cas10 variants under otherwise identical conditions. For metal‐dependence assays, EDTA was added prior to reaction initiation. For buffer‐dependence experiments, Tris was replaced with alternative buffers lacking primary alcohol groups while maintaining comparable pH and ionic strength.

### Radiolabeled TLC analysis

Reaction products were analyzed by thin‐layer chromatography (TLC) using radiolabeled nucleotides, as previously described for Cas10‐mediated ATP processing [7]. For TLC assays, reactions were assembled as described above and contained [α‐^32^P]ATP as the ATP substrate. Reaction mixtures were directly loaded onto polyethyleneimine (PEI)‐cellulose TLC plates.

TLC plates were developed in activation buffer consisting of 0.8 M acetic acid, 20% (v/v) ethanol, and 0.5 M LiCl. After development, the plates were air‐dried and radioactive signals were detected by autoradiography. Radiolabeled ATP standards were prepared from [α‐^32^P]ATP. Radiolabeled ADP was generated by incubating [α‐^32^P]ATP under kinase reaction conditions at 37 °C for 30 min, and radiolabeled AMP was prepared by heat treatment of ATP at 95 °C overnight, as described previously [7]. All standards were analyzed alongside experimental samples on the same TLC plate. All experiments involving radioactive materials were performed in accordance with institutional radiation safety regulations.

### High‐performance liquid chromatography

Reaction mixtures were analyzed by reverse‐phase high‐performance liquid chromatography using Shimadzu Prominence HPLC system (LC‐20AT) equipped with a UV detector and a Supelco Ascentis^®^ C18 column (5 μm, 4.6 × 250 mm, part no. 58348), following established procedures for separation of Cas10‐derived nucleotide products [7, 8, 9]. Separations were performed at 28 °C with a flow rate of 1.3 mL·min^−1^. The mobile phases consisted of eluent A (0.1 m KH_2_PO_4_, pH 4.6) and eluent B (eluent A:methanol, 90 : 10, v/v). A linear gradient program was applied as follows: 0–9 min, 0% B; 9–15 min, linear increase to 25% B; 15–17.5 min, linear increase to 90% B; 17.5–19 min, increase to 100% B; 19–23 min, held at 100% B for column washing; and 23–24 min, return to 0% B for re‐equilibration. The total run time was 24 min. Elution was monitored at 254 nm, and retention times were compared with those of nucleotide standards (ATP, ADP, AMP). Fractions corresponding to newly observed peaks were collected for mass spectrometric analysis. Since phosphate buffers interfere with MS analysis, similar experiments were conducted under MS‐compatible buffer conditions. Nevertheless, under phosphate‐free conditions, the newly observed peaks were not well separated chromatographically from the reaction mixture, although the product signal was still detected in the MS spectra. Due to the narrow elution window of the target peaks during online LC–MS analysis, which limited detailed characterization, the product fractions were collected separately. These fractions were subsequently analyzed by MSⁿ via direct infusion at a low flow rate to allow extended analysis time.

### Mass spectrometry and tandem MS analysis

Collected HPLC fractions were analyzed by direct infusion using an LTQ‐Orbitrap Elite (Thermo Fisher Scientific, Waltham, MA, USA) mass spectrometer equipped with a dual‐pressure linear ion trap and an Orbitrap analyzer. A dual‐pressure linear ion trap analyzer was used to acquire MS^n^ fragmentation data for structural characterization of the samples. Fragmentation was performed in negative ion mode using collision‐induced dissociation. Samples were introduced by syringe infusion with a flow rate of 1 μL·min^−1^ via a 20 μm ID tip installed on the EASY‐Spray™ source (P/N ES081; Thermo Fisher Scientific). The system was operated without sheath or auxiliary gas. The spray voltage was set to 1.9 kV. The ion transfer tube was maintained at a temperature of 320 °C. LTQ data were acquired with normal resolution. The isolation window was set to 1–4 Th according to ion intensity and MS^n^ level. Fragment ions were assigned based on expected AMP‐ and Tris‐derived fragments.

## Results

### Isolated Cas10 generates an ATP‐derived product distinct from canonical nucleotides

**Fig. 2:**
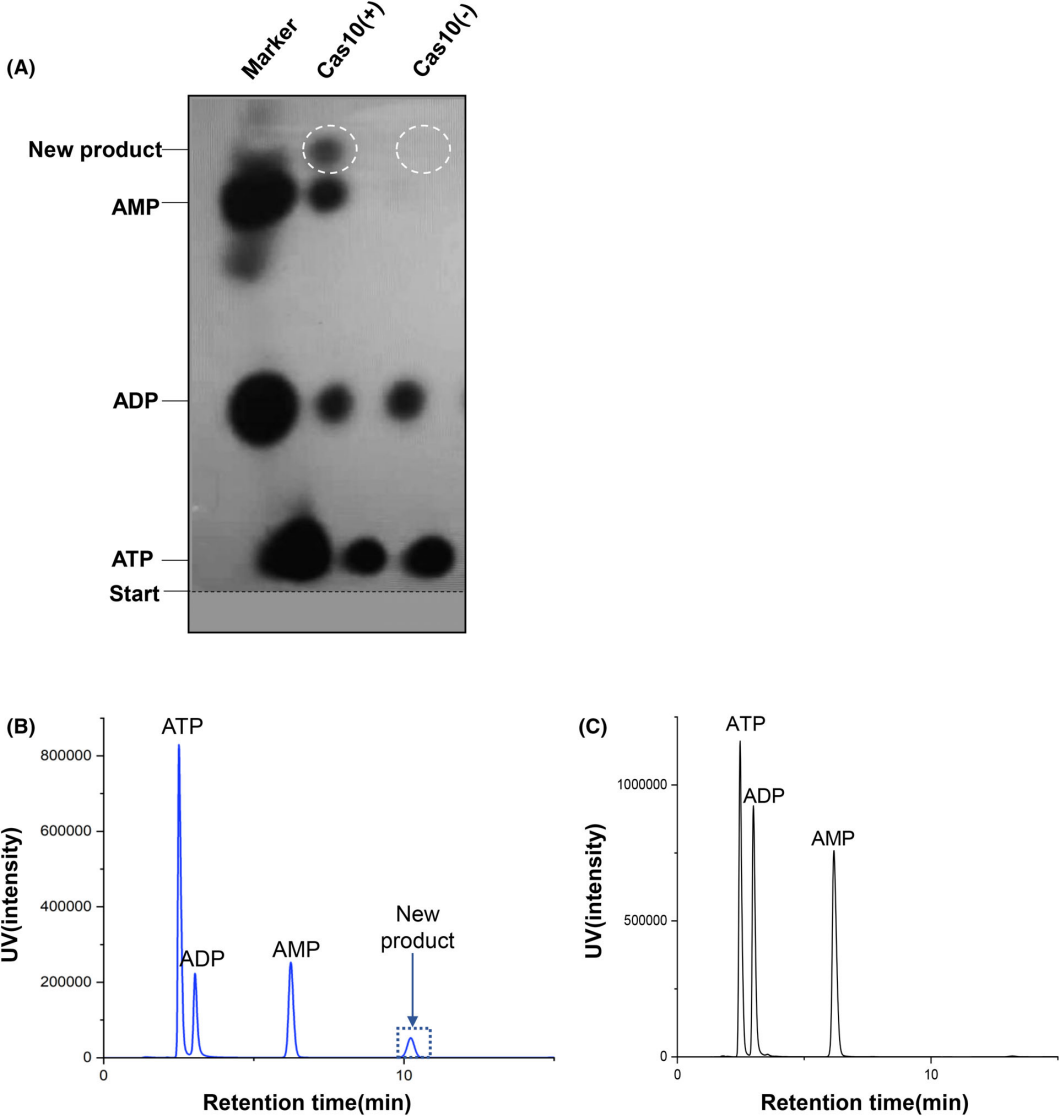
Detection of an ATP‐derived product generated by isolated Cas10. (A) TLC analysis of reaction products formed upon incubation of purified *T. onnurineus* Cas10 with ATP. An additional spot with mobility distinct from ATP, ADP, and AMP was observed in the presence of Cas10. (B) HPLC analysis of the same reaction mixtures, showing an additional peak with a retention time of 10.5 min that does not correspond to known nucleotide standards. Detection was performed using HPLC‐UV at 254 nm. (C) HPLC chromatograms of ATP, ADP, and AMP standards used as retention time references, recorded using HPLC‐UV detection at 254 nm.

To examine the intrinsic catalytic activity of Cas10, purified Cas10 from *T. onnurineus* was incubated with ATP under standard *in vitro* reaction conditions. Reaction products were first analyzed by thin‐layer chromatography (TLC) alongside nucleotide standards. In reactions containing Cas10, an additional spot was consistently observed that migrated at a position distinct from ATP, ADP, or AMP, whereas no such product was detected in enzyme‐free control reactions (Fig. 2A). The appearance of this additional species was dependent on the presence of Cas10 and was not observed upon incubation of ATP alone, indicating that it did not arise from spontaneous ATP degradation under the assay conditions.

Consistent with the TLC results, high‐performance liquid chromatography (HPLC) analysis monitored at 254 nm revealed an additional peak with a retention time of 10.5 min (Fig. 2B), which did not correspond to any known nucleotide standards, including ATP, ADP, or AMP (Fig. 2C). The formation of this peak was reproducible across independent experiments performed under identical reaction conditions and increased with prolonged incubation time. The distinct chromatographic behavior observed by both TLC and HPLC suggested the formation of a chemically modified ATP‐derived product rather than a known ATP hydrolysis intermediate. Together, these observations indicate that isolated Cas10 generates an ATP‐derived product distinct from the canonical cyclic oligoadenylates (cOA3–cOA6) synthesized by activated type III CRISPR–Csm complexes [7, 8, 9, 12].

### Mass spectrometric identification of the Cas10‐dependent product as AMP‐O‐Tris

**Fig. 3:**
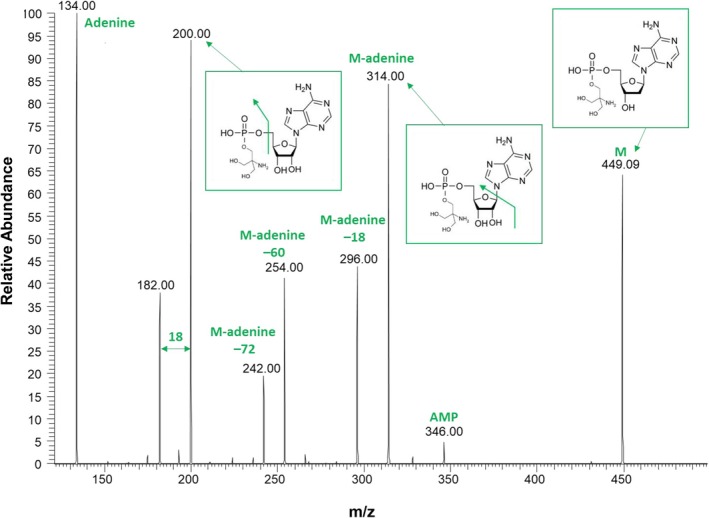
MS^n^ spectrum of the Cas10‐dependent product. The precursor ion at m/z 449.09 was subjected to MS^n^ fragmentation in ion trap mode using collision‐induced dissociation. Major fragment ions corresponding to AMP‐ and Tris‐derived moieties are indicated in the spectrum.

To determine the chemical identity of the newly observed product, the corresponding HPLC fraction was collected and subjected to mass spectrometric analysis. High‐resolution MS revealed a prominent ion species with a mass consistent with the formation of an AMP‐containing adduct. To confirm the structure of the product, MS^n^ analysis was performed by selecting the precursor ion at m/z 449.09, using collision‐induced dissociation (CID) with a collision energy of 30. Subsequent MS^n^ analysis produced characteristic fragment ions corresponding to the AMP moiety together with fragments derived from Tris (Fig. 3).

Notably, the observed fragmentation pattern matched that previously reported for AMP‐O‐Tris, an adenylylated Tris species formed in nonenzymatic ATP reactions [20]. Based on accurate mass measurements and diagnostic fragmentation profiles, the Cas10‐dependent product was unambiguously identified as AMP‐O‐Tris (Fig. 3).

### AMP‐O‐Tris formation depends on Cas10 catalytic activity

**Fig. 4:**
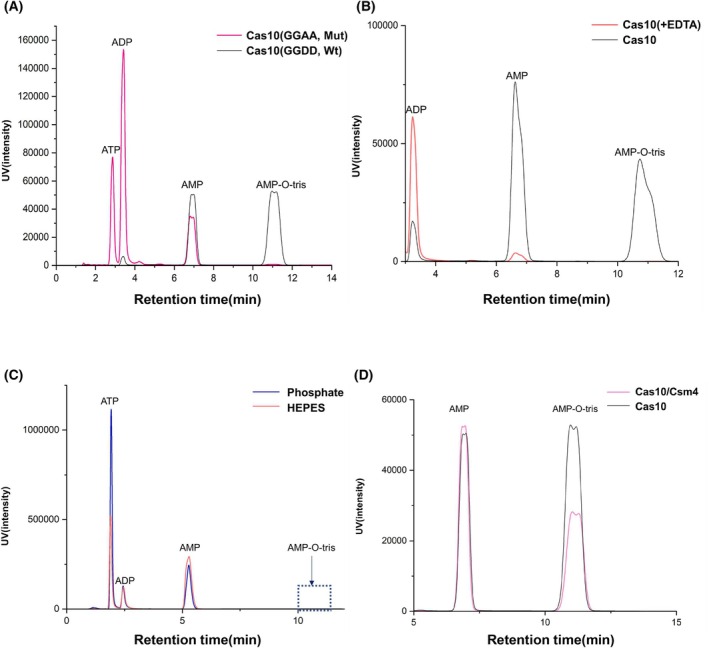
Representative HPLC chromatograms of ATP‐derived products generated by Cas10 under different reaction and assembly conditions. (A) Catalytically active Cas10 versus the inactive GGAA variant. AMP‐O‐Tris formation was observed only with active Cas10. (B) Loss of AMP‐O‐Tris formation in the presence of metal ion chelators such as EDTA, indicating a requirement for divalent metal ions. (C) AMP‐O‐Tris formation was abolished when Tris was replaced with buffers lacking primary alcohol groups. (D) AMP‐O‐Tris formation was also detected in reactions containing the Cas10–Csm4 subcomplex.

To assess whether AMP‐O‐Tris formation required Cas10 catalytic activity, control reactions were performed either in the absence of enzyme or using a catalytically inactive Cas10 variant carrying mutations in the conserved GGDD motif (GGAA) under otherwise identical conditions. No AMP‐O‐Tris signal was detected by mass spectrometry in reactions lacking Cas10, indicating that the product does not arise from spontaneous chemical reactions of ATP under the assay conditions. Likewise, reactions containing the GGAA variant failed to produce detectable AMP‐O‐Tris, despite comparable protein concentrations and incubation times (Fig. 4A).

In contrast, reactions containing catalytically active Cas10 consistently yielded AMP‐O‐Tris. In addition, AMP‐O‐Tris formation was abolished in the presence of metal ion chelators, indicating a strict requirement for divalent metal ions in the reaction (Fig. 4B). These results demonstrate that AMP‐O‐Tris formation depends on the catalytic machinery of Cas10 and on metal ion coordination and arises from enzyme‐mediated chemistry rather than nonspecific ATP reactivity.

### AMP‐O‐Tris formation occurs under isolated Cas10 reaction conditions

AMP‐O‐Tris formation was observed under reaction conditions containing purified Cas10, ATP, and Tris‐based buffer. When Tris was replaced with alternative buffers lacking primary alcohol groups, AMP‐O‐Tris formation was abolished, indicating that a buffer‐derived nucleophile directly participates in product formation (Fig. 4C). This buffer dependence further supports the interpretation that the observed product arises from a noncanonical adenylylation reaction involving an exogenous nucleophile.

AMP‐O‐Tris formation was also detected in reactions containing the Cas10–Csm4 subcomplex (Fig. 4D), indicating that partial complex assembly is insufficient to fully suppress this noncanonical activity. Together, these observations show that isolated Cas10 is sufficient to catalyze AMP‐O‐Tris formation under *in vitro* conditions and that this reaction occurs independently of full Csm complex assembly or target RNA binding.

## Discussion

In this study, we demonstrate that isolated Cas10 from *T. onnurineus* catalyzes a noncanonical ATP‐dependent adenylylation reaction, generating AMP‐O‐Tris under standard *in vitro* conditions. The strict dependence of this reaction on the conserved GGDD catalytic motif and on divalent metal ions indicates that AMP‐O‐Tris formation is mediated by the same catalytic machinery responsible for canonical cyclic oligoadenylate (cOA) synthesis [7, 8, 9]. These results reveal that, when removed from its native multiprotein context, Cas10 exhibits relaxed nucleophile selectivity, allowing small exogenous molecules to intercept adenylated intermediates during ATP turnover.

The formation of AMP‐O‐Tris provides insight into the regulatory role of effector complex assembly in type III CRISPR–Csm systems. Rather than simply activating Cas10, assembly of the Csm complex appears to constrain the chemical space accessible to the Cas10 active site, enforcing productive cOA synthesis while suppressing nonproductive side reactions. Interactions with accessory subunits and target RNA are therefore likely to limit solvent access and precisely position physiological nucleophiles, ensuring efficient coupling of ATP consumption to second‐messenger generation *in vivo* [11, 16, 17].

These findings provide mechanistic insights relevant to *in vitro* studies of type III CRISPR systems. Cas10‐mediated ATP turnover is increasingly utilized in CRISPR‐based diagnostic platforms that rely on cOA‐dependent activation of CARF‐domain effectors for signal amplification [19]. Our results suggest that, under certain reaction conditions, ATP may also be diverted into noncanonical adenylylation reactions, particularly in tris‐containing buffers or when Cas10 is not fully assembled within the effector complex, which could potentially influence ATP availability for productive cOA synthesis and may affect reaction efficiency or background activity *in vitro*. These observations highlight the importance of reaction conditions, including buffer composition and enzyme assembly state, when designing and interpreting experiments involving type III CRISPR systems.

Beyond technical considerations, the observed catalytic behavior of isolated Cas10 raises intriguing evolutionary implications. Although Cas10 activity is normally restricted to assembled CRISPR–Csm complexes, transient dissociation events, incomplete assembly, or selective degradation of accessory subunits could liberate catalytically active Cas10 domains. In such cases, unrestricted Cas10 activity could pose a risk of aberrant second‐messenger production. Our findings suggest that the abundance of primary alcohol–containing metabolites in cells may mitigate this risk: even if free Cas10 remains active, alcoholysis of ATP could potentially divert reactive intermediates into alcohol‐conjugated adenylylated products that are no longer competent as second messengers [20].

From this perspective, alcohol‐mediated diversion of Cas10 activity may function as an intrinsic chemical fail‐safe that neutralizes unintended signaling by orphan Cas10 enzymes. Alternatively, alcohol‐conjugated nucleotides generated by isolated Cas10 domains could participate in as‐yet‐unrecognized signaling or regulatory pathways. While speculative, this possibility broadens the conceptual framework of CRISPR‐associated nucleotide chemistry.

In summary, our work reveals a latent catalytic flexibility in Cas10 that is normally constrained by effector complex assembly. This flexibility underscores the importance of multiprotein organization in directing CRISPR enzyme chemistry toward productive immune signaling, while suggesting chemical and evolutionary mechanisms that limit unintended second‐messenger activity. These insights deepen our understanding of Cas10 regulation and inform the rational design of CRISPR‐based biochemical and diagnostic platforms.

## Conflict of interest

The authors declare no conflict of interest.

## Author contributions

The study was conceived by E‐JW and S‐JL, E‐JW, J‐HM, J‐HK, S‐RG, K‐HP, and S‐JL provided scientific and experimental suggestions. S‐JL and G‐SL performed the experiments and created the figures. The manuscript was written by S‐JL and E‐JW with input from all authors.

## Data Availability

All data generated or analyzed during this study are provided in this article.
